# Increasing prosthetic foot energy return affects whole-body mechanics during walking on level ground and slopes

**DOI:** 10.1038/s41598-018-23705-8

**Published:** 2018-03-29

**Authors:** W. Lee Childers, Kota Z. Takahashi

**Affiliations:** 1 School of Biological Sciences, College of Sciences, Georgia Institute of Technology, Atlanta, GA USA; 2 Department of Biomechanics, College of Education, University of Nebraska at Omaha, Omaha, NE USA

## Abstract

Prosthetic feet are designed to store energy during early stance and then release a portion of that energy during late stance. The usefulness of providing more energy return depends on whether or not that energy transfers up the lower limb to aid in whole body propulsion. This research examined how increasing prosthetic foot energy return affected walking mechanics across various slopes. Five people with a uni-lateral transtibial amputation walked on an instrumented treadmill at 1.1 m/s for three conditions (level ground, +7.5°, −7.5°) while wearing a prosthetic foot with a novel linkage system and a traditional energy storage and return foot. The novel foot demonstrated greater range of motion (p = 0.0012), and returned more energy (p = 0.023) compared to the traditional foot. The increased energy correlated with an increase in center of mass (CoM) energy change during propulsion from the prosthetic limb (p = 0.012), and the increased prosthetic limb propulsion correlated to a decrease in CoM energy change (i.e., collision) on the sound limb (p < 0.001). These data indicate that this novel foot was able to return more energy than a traditional prosthetic foot and that this additional energy was used to increase whole body propulsion.

## Introduction

A uni-lateral transtibial amputation imposes significant challenges for locomotion. The missing biological systems (part of the tibia and the foot/ankle complex) are replaced with a prosthesis. This prosthesis consists of a prosthetic socket shaped to conform to the patient’s residual limb, a method to suspend, or attach, the prosthesis to the residual limb, a prosthetic foot, and a connector between the foot and the socket. The prosthesis is an imperfect replacement for the biological system it was designed to replace. The connection between the human and the prosthesis occurs through the soft tissues of the residual limb and the low stiffness^1^ of these tissues allow for substantial movement and energy absorption^2^. The majority of prosthetic feet available for clinical use are passive and can only return a portion of the energy they absorb when deformed during gait^3,4^. In contrast, the biological foot/ankle complex is under active control of the neuromuscular system and can typically produce more energy via muscle tendon unit dynamics^5^ than a passive prosthetic foot for push-off during late stance^6^.

Consequences of reduced push-off from the amputated limb include asymmetric gait^3,6–9^, and increased loading on the sound limb^6,8,9^. Furthermore, greater sound limb loading, as indicated by increases in magnitude of the first peak of the vertical ground reaction force (GRF), the slope of the vertical GRF during early stance, or first peak of the external knee adduction moment (EKAM), may play a significant role in the development joint osteoarthritis^10–12^, a condition that this population may be at higher risk to develop^13–18^. Biomechanical mechanisms reported from dynamic walking models and human walking experiments may explain the connection between low push-off on the amputated limb and increased loading on the sound limb^19–21^. There is an inverse relationship between positive work performed by the trailing limb to redirect the whole body center of mass (CoM) and the negative work performed on the leading limb^20^. When the trailing limb, e.g. amputated limb, does not perform sufficient work during late stance phase to help redirect the CoM, the leading limb collides with ground at a higher and downward directed velocity^22^. This results in increasing the amount of negative work done by the leading limb, i.e. sound limb, during collision with the ground, and thus increases the amount of energy that must be absorbed by eccentric contraction of muscles, soft tissues, and at the joints themselves^23^. Therefore, reduction of negative work performed by the sound limb during collision may help reduce joint loading and reduce risk of developing knee osteoarthritis.

Providing more energy from the prosthetic foot may leverage dynamic walking mechanisms to help reduce sound limb loading^8,24^. Increasing the energy return from a passive prosthetic foot to higher than traditional levels of push-off (but lower than biological foot/ankle) reduces the first peak of the vertical GRF and the first peak of the EKAM in the sound limb^9^. Increasing the energy generation via powered prosthetic ankle/foot system to levels of biological ankle reduced CoM energy change during collision by the sound limb in one study^24^ but not others^25,26^. Esposito *et al*.^25^ demonstrated no change in sound limb collision work when walking on level ground. Quesada *et al*.^26^ demonstrated a reduction in sound limb collision work while the prosthetic foot delivered lower than biological levels of ankle power, but this benefit tapered to no change in collision work for ankle power at or beyond biological levels. Why energy from the prosthetic foot does not always relate to reducing sound limb collision work may be related to energy dissipated at the limb socket interface, motor strategies that may inhibit energy transfer up the kinematic chain of the lower limb to affect CoM mechanics^26^, or by not challenging the motor system enough to elicit a change^27^.

Walking uphill and downhill may offer a method to challenge the motor system^25,27–29^. The lower limbs must overall absorb energy and limit propulsion from the trailing limb during downhill walking^30^. In contrast, uphill walking has a reverse strategy in that the motor system must produce net positive energy to propel the CoM against gravity and would necessitate additional propulsion from the trailing limb^30^. Walking uphill and downhill is challenging for people with amputation due to the limited range of motion (ROM) provided by the prosthetic foot and likely the limited push-off provided by a passive prosthetic foot^31–33^. Passive prosthetic feet must rely on their stiffness to provide the ROM necessary to conform to sloped terrains. The lower the stiffness, the better the foot can conform to a slope but this comes at the expense of work ratio (ratio of energy returned to energy absorbed) and lower energy return for propulsion^3^. This creates a challenging set of criteria for designers of passive prosthetic feet. The prosthetic foot needs to be deformable in order to store and return energy, but the more a prosthetic foot can deform with damped materials, the less able it is to deliver energy for propulsion. This may be advantageous for downhill walking where increased deformation and high damping may be desirable to minimize trailing limb propulsion^30^. Yet, this would not be advantageous for level ground or uphill walking when increasing propulsion from the trailing limb would be desirable^30^. Therefore, walking uphill and downhill not only challenges the motor system, but creates a challenging environment to test novel prosthetic foot designs.

**Figure 1:**
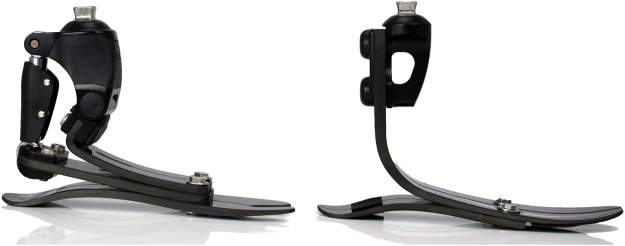
The Pro-Flex foot (left) has a linkage system on the upper portion of the foot that is designed to non-linearly load the heel and forefoot sections. This is a departure from conventional energy storage and return type prosthetic feet like the Vari-Flex (right).

The purpose of this study was to examine how prosthetic designs intended to increase energy return affects mechanics of walking across various slopes (level, uphill, and downhill). We utilized a conventional energy-storing-and-returning (ESR) foot (Össur Vari-Flex), and a novel ESR prosthetic foot (Össur Pro-Flex) (Fig. 1). The Pro-Flex was designed to increase the conformability of the prosthetic foot to slopes and increase the amount of energy returned for propulsion^34^. By comparing the two types of prosthetic feet, our aim was to identify biomechanical effects of prosthetic energy return on sound limb loading across level, uphill and downhill terrains. We hypothesized that: 1) range of motion will be greater with the Pro-Flex foot than the Vari-Flex foot, 2) the increased RoM from the Pro-Flex foot will contribute to greater energy storage and return than the Vari-Flex foot, 3) the increased energy return during push-off from the Pro-Flex will increase CoM energy change by the amputated limb during propulsion, and 4) the enhanced propulsion from the Pro-Flex foot will reduce loads on the sound limb.

## Results

### Range of motion was increased with the Pro-Flex foot

**Figure 2:**
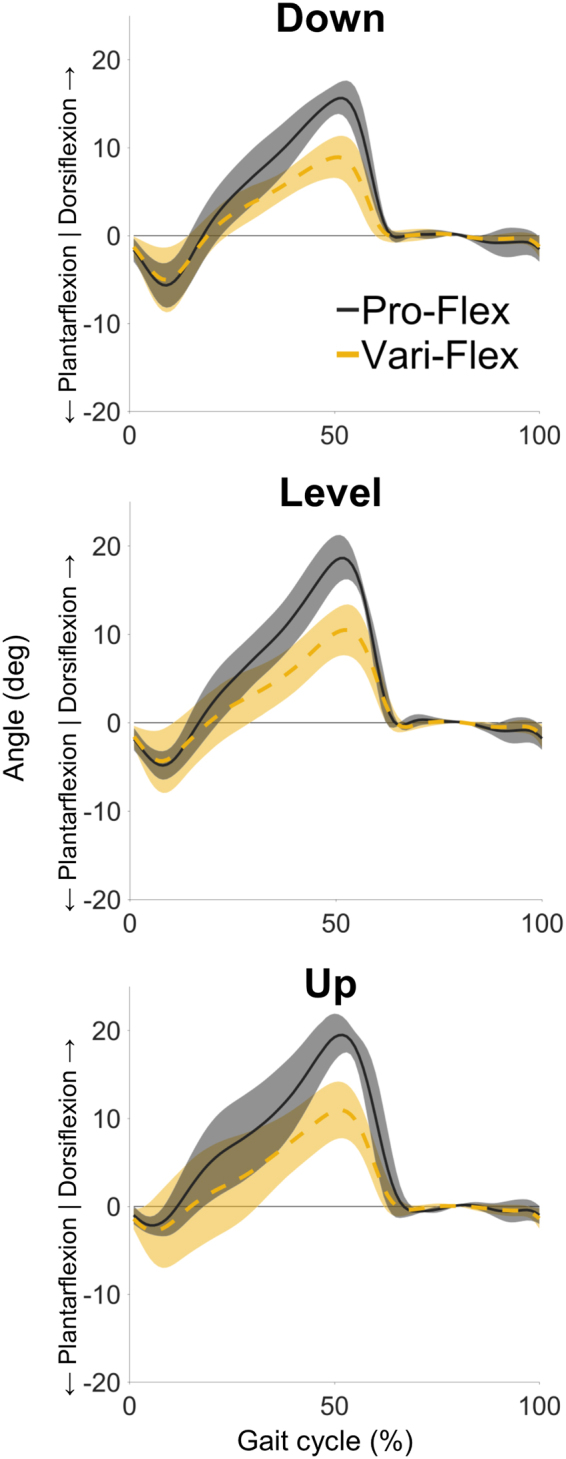
Angle between the prosthetic shank and foot segments demonstrate that the Pro-Flex foot (black solid line) was able to dorsiflex more throughout stance phase than the Vari-Flex foot (yellow dashed line) across all conditions. Shaded regions represent ± one standard deviation. The steeper slope of these lines indicate the Pro-Flex foot was able to more rapidly dorsiflex and this may have helped to increase the amount of energy that was stored in the forefoot.

There was a significant effect of foot for peak dorsiflexion angle (p = 0.003), and range of motion between the shank and foot segments (p = 0.012) without a significant interaction effect (p = 0.632), and these results coincided with large effect sizes (Table 1). The greater range of motion demonstrated by the Pro-Flex foot was more strongly correlated with peak dorsiflexion angle (R = 0.840, p < 0.001) than peak plantarflexion angle (R = 0.597, p < 0.001). This was associated with an increased angular velocity of the shank rotating over the prosthetic foot for the Pro-Flex foot condition (Fig. 2).

**Table 1:** Main results for the Pro-Flex and Vari-Flex feet at a treadmill speed of 1.1 m/s across downhill (−7.5°), level (0°), and uphill (+7.5°) terrains.

Variable	Foot	Terrain			Effect of terrain	Effect of foot	Interaction effect
		Down	Level	Up			
Peak dorsiflexion angle (deg)	Pro-Flex*	15.9 ± 2.1	18.8 ± 2.7	20 ± 2.3	F = 5.896	F = 45.363	F = 5.839
					*p* = 0.027	*p* = 0.003	*p* = 0.027
	Vari-Flex	9.2 ± 2.9	10.6 ± 3.2	11.1 ± 3.6	*η*_*p*_^2^ = 0.706	*η*_*p*_^2^ = 0.919	*η*_*p*_^2^ = 0.593
					1 − β = 0.715	1 − β = 0.998	1 − β = 0.711
Peak plantarflexion angle (deg)	Pro-Flex	5.8 ± 2.7	4.9 ± 1.8	2.4 ± 1.3	F = 14.915	F = 0.001	F = 4.534
					*p* = 0.017	*p* = 0.991	*p* = 0.091
	Vari-Flex	5.1 ± 4.1	4.6 ± 3.9	3.4 ± 4.3	*η*_*p*_^2^ = 0.789	*η*_*p*_^2^ = 0.001	*η*_*p*_^2^ = 0.531
					1 − β = 0.830	1 − β = 0.050	1 − β = 0.405
Range of motion between shank and foot segments (deg)	Pro-Flex*	21.7 ± 2.7	23.7 ± 3.1	22.4 ± 2.3	F = 1.626	F = 19.315	F = 0.487
					*p* = 0.255	*p* = 0.012	*p* = 0.632
	Vari-Flex	14.2 ± 6.6	15.2 ± 6	14.5 ± 5.8	*η*_*p*_^2^ = 0.289	*η*_*p*_^2^ = 0.828	*η*_*p*_^2^ = 0.109
					1 − β = 0.249	1 − β = 0.900	1 − β = 0.105
Energy stored in the prosthetic foot (J/kg)	Pro-Flex*	−0.24 ± 0.05	−0.23 ± 0.02	−0.24 ± 0.05	F = 0.103	F = 13.130	F = 0.301
					*p* = 0.903	*p* = 0.022	*p* = 0.748
	Vari-Flex	−0.18 ± 0.03	−0.18 ± 0.02	−0.17 ± 0.03	*η*_*p*_^2^ = 0.025	*η*_*p*_^2^ = 0.766	*η*_*p*_^2^ = 0.070
					1 − β = 0.061	1 − β = 0.772	1 − β = 0.083
Total energy returned by the prosthetic foot (J/kg)	Pro-Flex*	0.2±0.09	0.16 ± 0.01	0.2 ± 0.04	F = 2.111	F = 8.298	F = 0.147
					*p* = 0.184	*p* = 0.045	*p* = 0.865
	Vari-Flex	0.15 ± 0.01	0.11 ± 0.02	0.14 ± 0.05	*η*_*p*_^2^ = 0.345	*η*_*p*_^2^ = 0.675	*η*_*p*_^2^ = 0.035
					1 − β = 0.313	1 − β = 0.586	1 − β = 0.066
Energy returned by the prosthetic foot during push-off	Pro-Flex*	0.18 ± 0.08	0.16 ± 0.01	0.19 ± 0.04	F = 1.627	F = 12.877	F = 0.083
					*p* = 0.255	*p* = 0.023	*p* = 0.922
	Vari-Flex	0.13 ± 0.03	0.1 ± 0.01	0.12 ± 0.05	*η*_*p*_^2^ = 0.289	*η*_*p*_^2^ = 0.763	*η*_*p*_^2^ = 0.020
					1 − β = 0.249	1 − β = 0.764	1 − β = 0.059
Work ratio of the prosthetic foot (J/J)	Pro-Flex	0.78 ± 0.22	0.71 ± 0.06	0.87 ± 0.18	F = 3.640	F = 0.355	F = 0.992
					*p* = 0.075	*p* = 0.584	*p* = 0.412
	Vari-Flex	0.86 ± 0.16	0.60 ± 0.13	0.79 ± 0.25	*η*_*p*_^2^ = 0.476	*η*_*p*_^2^ = 0.081	*η*_*p*_^2^ = 0.199
					1 − β = 0.501	1 − β = 0.075	1 − β = 0.167
Energy delivered by the foot/ankle during push-off by the sound limb (J/kg)	Pro-Flex	0.13 ± 0.06^†^	0.20 ± 0.06^‡^	0.39 ± 0.09 ^†,‡^	F = 27.308	F = 0.784	F = 0.812
					*p* < 0.001	*p* = 0.426	*p* = 0.477
	Vari-Flex	0.12 ± 0.04^†^	0.17 ± 0.05^‡^	0.4 ± 0.11 ^†,‡^	*η*_*p*_^2^ = 0.872	*η*_*p*_^2^ = 0.164	*η*_*p*_^2^ = 0.169
					1 − β = 0.999	1 − β = 0.107	1 − β = 0.144
CoM energy change during collision by the amputated limb (J/kg)	Pro-Flex	−0.11 ± 0.08	−0.09 ± 0.05	−0.09 ± 0.06	F = 0.243	F = 1.954	F = 0.212
					*p* = 0.656	*p* = 0.235	*p* = 0.713
	Vari-Flex	−0.10 ± 0.08	−0.08 ± 0.04	−0.07 ± 0.04	*η*_*p*_^2^ = 0.057	*η*_*p*_^2^ = 0.328	*η*_*p*_^2^ = 0.050
					1 − β = 0.068	1 − β = 0.192	1 − β = 0.067
CoM energy change during propulsion by the amputated limb (J/kg)	Pro-Flex*	0.10 ± 0.03^†^	0.15 ± 0.02^‡^	0.10 ± 0.02^†,‡^	F = 5.937	F = 352.81	F = 0.137
					*p* = 0.026	*p* < 0.001	*p* = 0.874
	Vari-Flex	0.07 ± 0.05^†^	0.12 ± 0.03^‡^	0.07 ± 0.02^†,‡^	*η*_*p*_^2^ = 0.597	*η*_*p*_^2^ = 0.989	*η*_*p*_^2^ = 0.033
					1 − β = 0.718	1 − β = 0.999	1 − β = 0.065
CoM energy change during collision by the sound limb (J/kg)	Pro-Flex	−0.31 ± 0.1	−0.14 ± 0.06	−0.15 ± 0.06	F = 6.178	F = 0.663	F = 0.378
					*p* = 0.065	*p* = 0.461	*p* = 0.602
	Vari-Flex	−0.34 ± 0.17	−0.14 ± 0.06	−0.18 ± 0.07	*η*_*p*_^2^ = 0.607	*η*_*p*_^2^ = 0.142	*η*_*p*_^2^ = 0.086
					1 − β = 0.486	1 − β = 0.098	1 − β = 0.080
CoM energy change during propulsion by the sound limb (J/kg)	Pro-Flex	0.09 ± 0.09	0.20 ± 0.07	0.25 ± 0.06^†,‡^	F = 26.529	F = 3.490	F = 4.102
					*p* < 0.001	*p* = 0.135	*p* = 0.059
	Vari-Flex	0.10 ± 0.08	0.18 ± 0.06	0.29 ± 0.07^†,‡^	*η*_*p*_^2^ = 0.869	*η*_*p*_^2^ = 0.466	*η*_*p*_^2^ = 0.506
					1 − β = 0.999	1 − β = 0.301	1 − β = 0.551
Magnitude of the first peak of the GRF on the sound limb (N/W)	Pro-Flex*	1.22 ± 0.14	0.98 ± 0.09	1.05 ± 0.06	F = 13.116	F = 7.332	F = 0.909
					*p* = 0.003	*p* = 0.054	*p* = 0.441
	Vari-Flex	1.27 ± 0.17	1.08 ± 0.06	1.08 ± 0.07	*η*_*p*_^2^ = 0.766	*η*_*p*_^2^ = 0.647	*η*_*p*_^2^ = 0.185
					1 − β = 0.969	1 − β = 0.537	1 − β = 0.157
Magnitude of the first peak of the knee adduction moment in the sound limb (Nm/kg)	Pro-Flex	0.5 ± 0.12	0.52 ± 0.19	0.37 ± 0.17	F = 3.982	F = 2.021	F = 1.235
					*p* = 0.063	*p* = 0.341	*p* = 0.341
	Vari-Flex	0.52 ± 0.13	0.50 ± 0.12	0.43 ± 0.17	*η*_*p*_^2^ = 0.499	*η*_*p*_^2^ = 0.236	*η*_*p*_^2^ = 0.236
					1 − β = 0.539	1 − β = 0.197	1 − β = 0.198

*Significant main effect between prosthetic feet. ^†^Significant difference from the level terrain. ^‡^Significant difference from the downhill terrain. Note, there are instances in which there was a significant effect of terrain yet the post hoc revealed no differences between terrains. Effect size was estimated using partial eta squared (η_p_^2^). Observed power was calculated as 1 − β.

### Energy return was greater with the Pro-Flex foot

**Figure 3:**
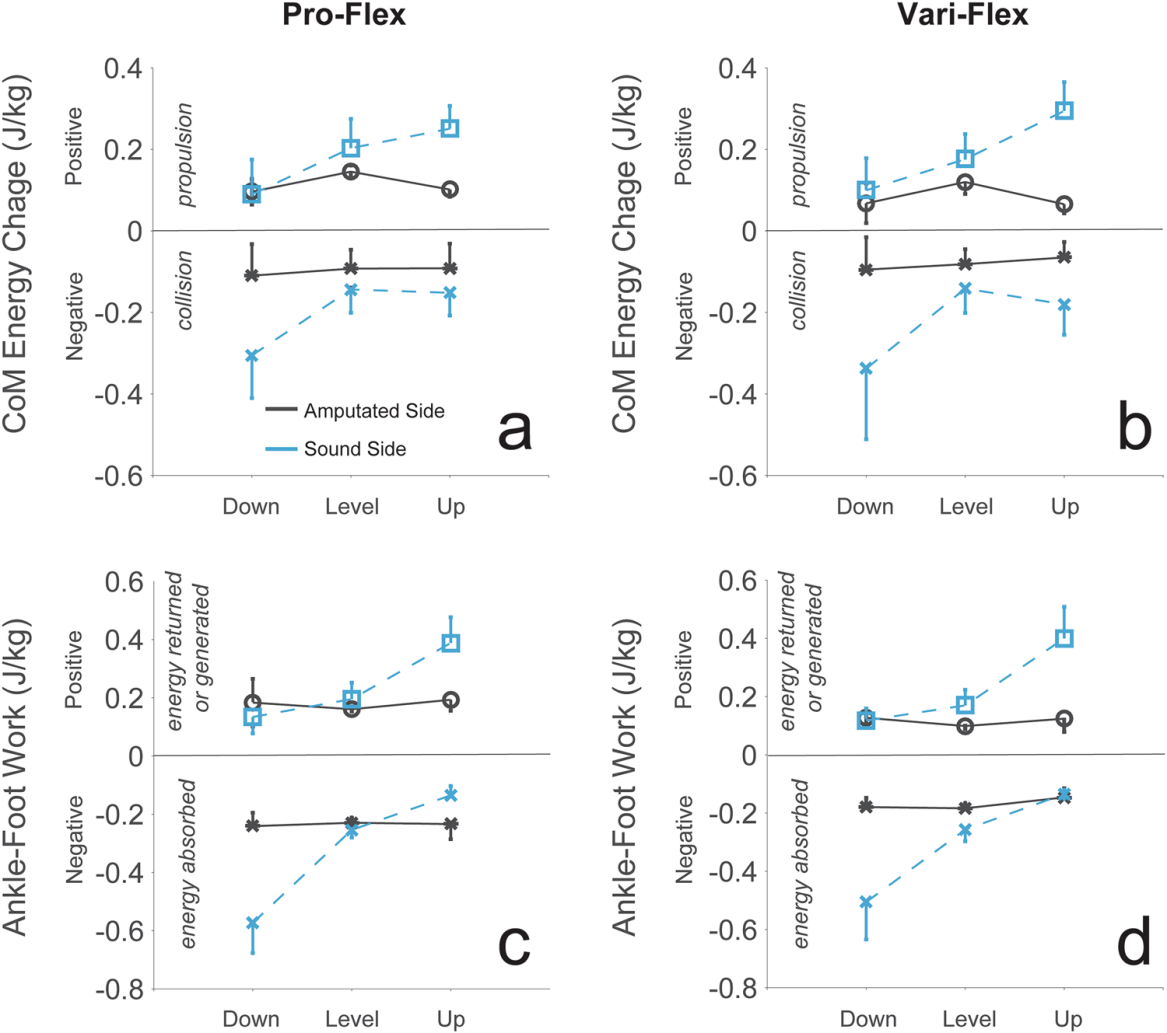
CoM energy change during propulsion (open symbols) and collision (closed symbols) for the Pro-Flex foot (panel a) and the Vari-Flex foot (panel b) compared with energy absorbed by the foot/ankle complex (closed symbols) and returned/generated (open symbols) by the Pro-Flex foot (panel c) and the Vari-Flex foot (panel d). Data trends for the amputated limb (solid black line) and the sound limb (dashed blue line) show that the storage and return of energy in the passive prosthetic is more constant than the sound limb across terrains, the Pro-Flex foot does return more energy than the Vari-Flex foot, and the CoM energy change during propulsion is larger with the Pro-Flex foot. Error bars represent ± one standard deviation.

The Pro-Flex foot demonstrated greater energy storage and return than the Vari-Flex foot (Fig. 3). The Pro-Flex foot stored more energy during stance than the Vari-Flex foot (p = 0.022), returned more energy (p = 0.045), more of that energy was delivered during push-off (p = 0.023), and these results occurred with large effect sizes and observed power (Table 1). The work ratios between the Pro-Flex foot and the Vari-Flex foot was not significantly different (p = 0.584) (Table 1). The increased dorsiflexion by the Pro-Flex foot correlated with the increased energy storage (R = 0.625, p < 0.001), energy returned (R = 0.438, p = 0.016), and energy returned during push-off (R = 0.547, p = 0.002).

### Energy return from the Pro-Flex foot was mostly lower than sound limb ankle-foot system

**Figure 4:**
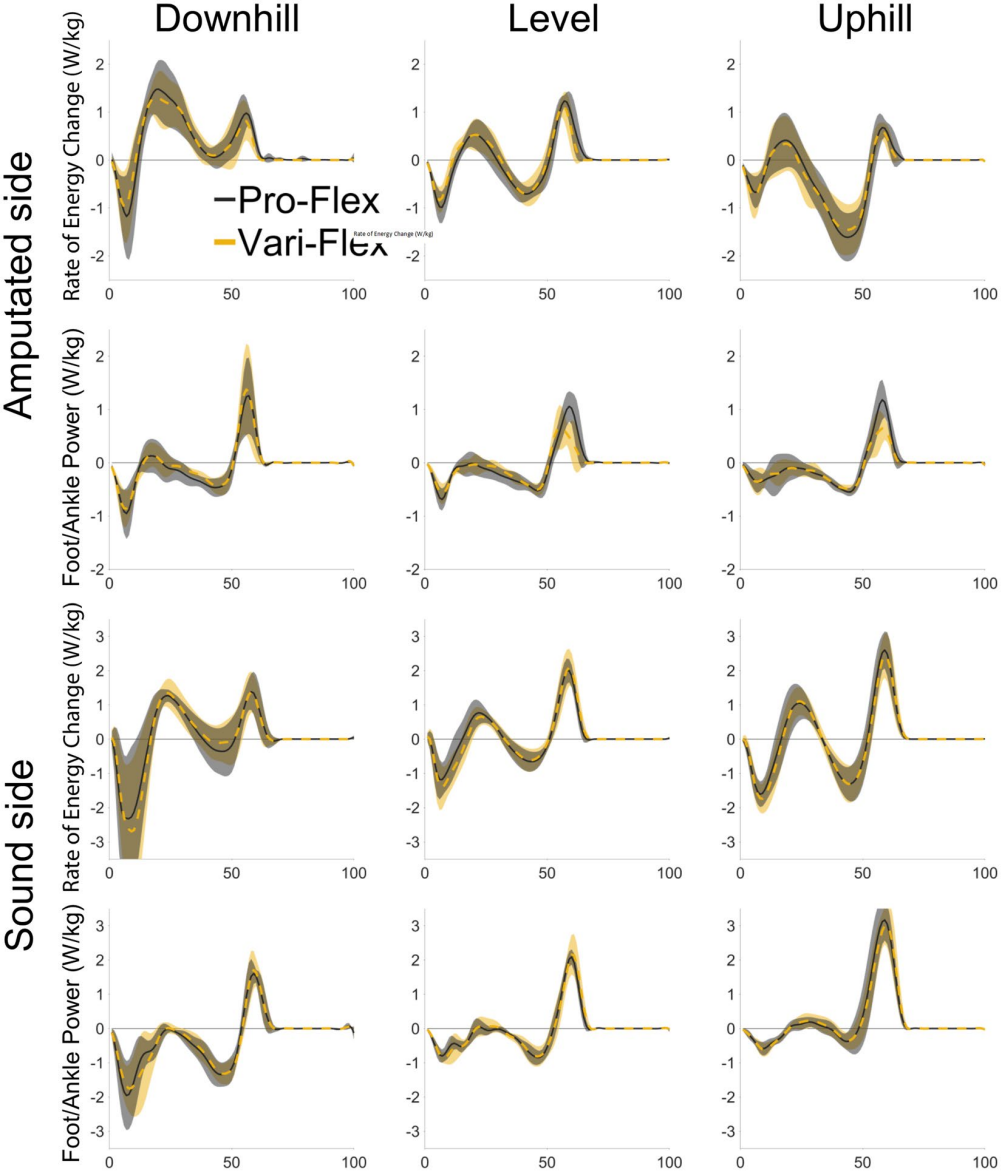
CoM rate of energy change (rows 1 & 3) and foot/ankle power (rows 2 & 4) throughout the gait cycle for downhill (column 1), level (column 2), and uphill (column 3) for the amputated limb (rows 1 & 2) and the sound limb (rows 3 & 4). The Pro-Flex foot (solid black line) demonstrated higher peak powers than the Vari-Flex foot (dashed yellow line) and this resulted in a trend to reduce the absorption of energy by the sound limb during the step-to-step transition.

There are some notable differences between the sound and amputated limbs. There was an overall significant difference in the energy returned (or generated) during push-off between the prosthetic foot and the sound ankle-foot system (p = 0.006). Pairwise comparisons demonstrated that the Pro-Flex foot did not return a significantly different amount of energy during push-off than the sound ankle-foot system during the downhill condition (p = 0.735) (Fig. 4). The ability of the Pro-Flex to deliver the same amount of energy for propulsion as the sound ankle-foot was because the sound ankle-foot reduced its output during the downhill condition. The amount of energy absorbed by the either prosthetic foot was significantly less than the sound ankle-foot (p < 0.001) (Fig. 3). The CoM negative energy change during collision performed by the amputated limb was also significantly less than the sound limb (p = 0.002), independent of the prosthetic foot used (Fig. 4).

### Energy from the Pro-Flex foot affected whole body center of mass mechanics

The CoM energy change during propulsion performed by the amputated limb was significantly increased with the Pro-Flex foot over the lower performing Vari-Flex foot (p < 0.001) with a large effect size and high observed power (Table 1). The increase in the amount of energy returned between the Pro-Flex foot and the Vari-Flex foot per subject and per condition was significantly correlated with the increase in CoM energy change during propulsion between the two feet (R = 0.650, p = 0.012).

### Reduction of loading on the sound limb was unclear

**Figure 5:**
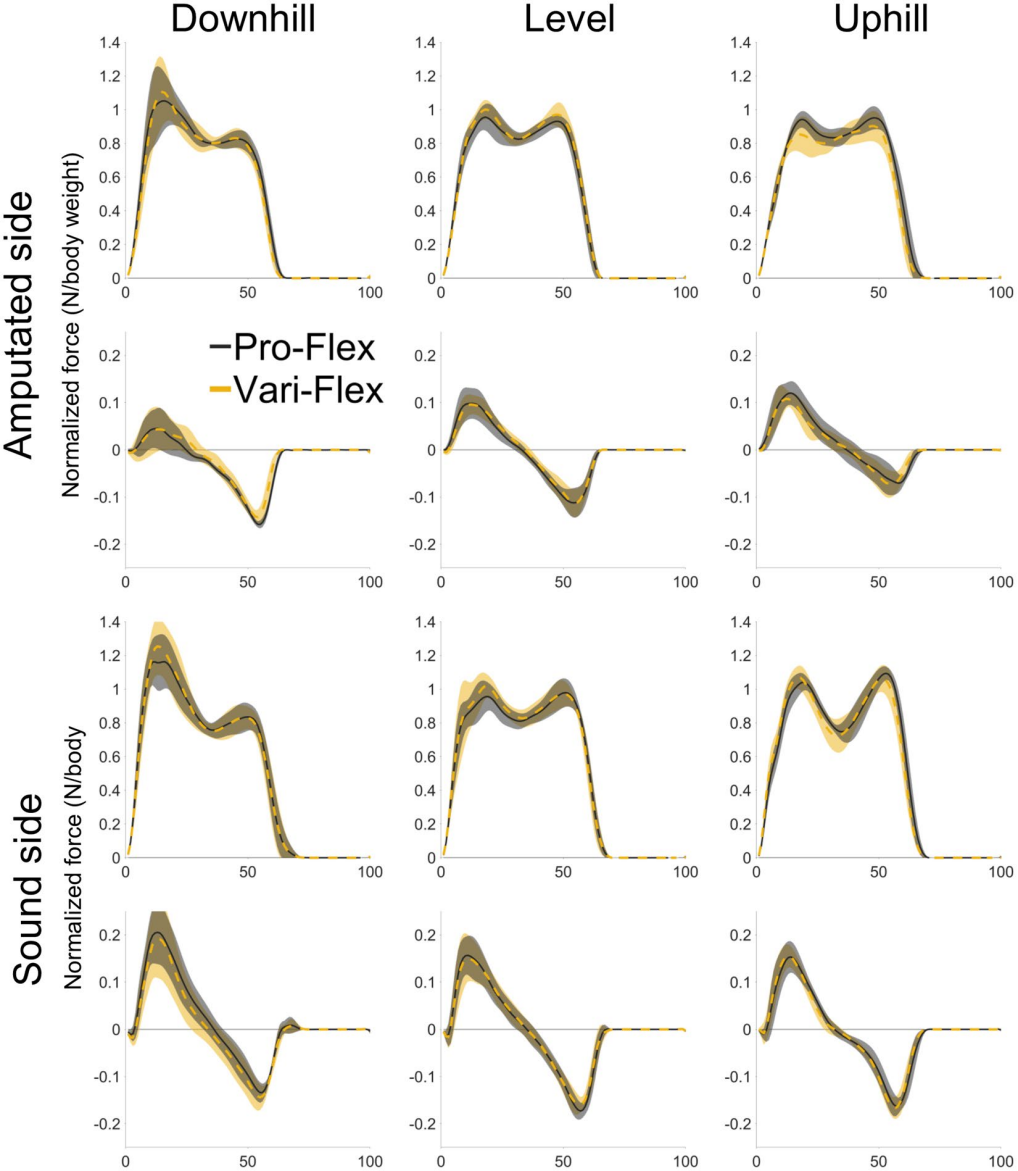
Vertical (rows 1 & 3) and horizontal (rows 2 & 4) components of the ground reaction force throughout the gait cycle for downhill (column 1), level (column 2), and uphill (column 3) for the amputated limb (rows 1 & 2) and the sound limb (rows 3 & 4). The Pro-Flex foot (solid black line) demonstrated a trend to reduce the first peak of the vertical ground reaction force on the sound limb compared to the Vari-Flex foot (dashed yellow line). There were no discernable differences between feet associated with lowering the loading rate and no changes in the horizontal components.

The Pro-Flex was unable to significantly reduce the first peak of the vertical component of the ground reaction force on the sound limb (p = 0.054) but this was associated with a moderate effect size (η_p_^2^ = 0.647) and low statistical power (1 − β = 0.537) (Table 1). The trend to reduce the magnitude of forces on the sound limb (Fig. 5) was one of many ways to measure loading on the sound limb. There were no differences between prosthetic feet regarding loading rate (Fig. 5). The magnitude of the peak knee adduction moment was also unaffected by the prosthetic foot (p = 0.341) but was also associated with low statistical power (1 − β = 0.197) (Table 1). There was a significant inverse correlation between CoM energy change during propulsion performed by the amputated limb and CoM energy change performed by the sound limb during collision (R = −0.611, p < 0.001). Despite this, there was no significant effect of foot on CoM energy change during collision performed by the sound limb (p = 0.461) and this was also associated with a lower effect size and observed power (Table 1).

## Discussion

The novel linkage system in the Pro-Flex foot enabled greater range of motion (Fig. 2) and delivered more energy during push-off than the more traditional energy storage and return Vari-Flex prosthetic foot (Fig. 3). The energy change of the CoM during propulsion was also increased with the Pro-Flex foot across all conditions (Fig. 3) and this correlated with a significant decrease in CoM energy change during collision on the sound limb.

The increased propulsion from the Pro-Flex foot was apparent across all conditions (Fig. 3). The Pro-Flex foot condition demonstrated greater range of motion, and more dorsiflexion than the Vari-Flex foot (Fig. 2). This allowed the forefoot to absorb more energy during late stance and subsequently return more energy (Fig. 4). The greater dorsiflexion from the Pro-Flex foot was likely facilitated by the mechanical design of the foot and the incorporation of the linkage system (Fig. 1). Passive elastic prosthetic feet work by being deformed to store elastic energy in the carbon fiber laminate structure and then allowing those structures to recoil and return the energy for propulsion during pre-swing. Deformation of a prosthetic foot results in eventual recoil and energy returned. The Pro-Flex foot was able to deform more than the Vari-Flex foot, utilize this deformation to better conform to the terrain, and subsequently recoil to return more energy during late stance.

The connection between increased range of motion and increased energy return during propulsion may not exist across all prosthetic feet. There are other prosthetic feet available that allow for increased range of motion and leverage that range of motion to better conform to slopes but do so in a different manner than presented here. The position of the long axis of the foot relative to the shank at any given point in the gait cycle represents a balance between the stiffness properties of the material, the damping properties of the material, the shape of the structure, the loads imposed on it via the environment, and how the knee and hip joint moments are coordinated by the prosthesis user. Therefore, equivalent ranges of motion are attainable with different designs of a prosthetic foot. For example, the Proprio foot (Össur EHF) is an adaptive ankle system with a servo motor that changes the angle of a low profile ESR foot. Range of motion provided by this system will be related to how the motor positions the foot and the deformation of the ESR foot. This combination does provide a large amount of adaptability to slopes and helps to reduce pressures between the prosthetic socket and the residual limb^35^. Yet, since the ESR portion of the prosthetic foot is not being deformed throughout the range of motion provided by the entire foot/ankle system, it is unlikely that this will result in greater propulsion than an ESR foot (like the Pro-Flex) that gains range of motion entirely through deformation of its carbon laminate. Likewise, energy returned during propulsion may not be as high in hydraulically controlled prosthetic ankle units or prosthetic feet made of fiberglass. The function of the hydraulic damper is to remove energy from the foot/ankle system in a way that allows for increased range of motion without the ability to return that energy for propulsion back out of the hydraulic system. Feet made of fiberglass are low stiffness but also come with high internal damping properties that should lower the work ratio of the foot and lower energy return for an equivalent deformation compared to a prosthetic foot made of carbon laminate. Therefore, the connection between prosthetic foot range of motion and energy return may not be generalizable to all feet due to the inherit interaction effects between the foot mechanical properties and how the prosthesis user is controlling the device. The ability of the Pro-Flex foot to have high amounts of range of motion and high energy return is due to the structure of this foot being deformed throughout its range of motion.

Analysis of whole body CoM mechanics was able to inform how well the energy being returned by the prosthetic foot was able to translate up the kinematic chain and be utilized to help propel the CoM. The increase in energy return between the Pro-Flex foot and the Vari-Flex foot (~0.06 J/kg) did correlate to an overall increase in CoM energy change (~0.02 J/kg) during propulsion for the amputated limb. An interesting part of these data was that the energy returned by the Pro-Flex foot and the Vari-Flex foot generally remained invariant across the different terrains, whereas the CoM energy change during propulsion on the amputated side did vary so that the level ground condition was higher than the downhill and uphill conditions (Fig. 3). Even though the human motor system did utilize the increase in energy return from the Pro-Flex foot and transfer it up the kinematic chain to the whole body CoM in a general sense, there was some terrain specific variation. The passive nature of an ESR foot means that its energy return is mainly influenced by the material properties and the loading/unloading characteristics^3,4,36,37^. The general invariability of energy returned by the prosthetic foot meant that in some conditions, (e.g. walking downhill) the prosthetic foot may be returning too much energy, while in other conditions (e.g. walking uphill), the prosthetic foot may not be returning enough energy. This created scenarios when the person’s motor system may want to dissipate and inhibit energy transfer from the prosthetic foot to the whole body CoM, such as during downhill walking (Fig. 4). In terrains where the increased energy return from the prosthetic foot would benefit CoM energy change (e.g. level ground and walking uphill), the increased energy return from the Pro-Flex foot could be transferred to the whole body CoM. However, our data remains too limited to distinguish the terrain specificity of how energy return from the prosthetic foot is transferred up the kinematic chain to the whole body CoM but creates an interesting topic for additional study.

These data were able to show promise that increasing energy return from a passive prosthetic foot would decrease sound limb loading. There was a significant inverse correlation between CoM energy change during propulsion performed by the amputated limb and CoM energy change performed by the sound limb during collision. This occurred despite the potential for altered strategies to dissipate energy return during downhill walking. However, the other data supporting that the additional energy return would reduce sound limb loading was difficult to demonstrate with these data. We used indirect estimations of sound limb loading that have been shown in previous literature to correlate with risk of developing knee osteoarthritis. The first peak of the external knee adduction moment correlates with a reduction in bone mineral density around the knee joint of the sound limb in people with transtibial amputation^12^ and correlates with medial compartment knee osteoarthritis severity^11^. Our data did not demonstrate a difference in the external knee adduction moment (Table 1) but this was associated with low statistical power meaning more subjects would have been necessary to draw a conclusion about this variable. There are also many ways a person may alter their gait to modulate the external knee adduction moment, e.g. increasing step width^38,39^ and lateral trunk lean^40^. The modulation of these unmeasured gait parameters may have contributed to the higher intersubject variability and low-statistical power and should be included in future studies interested in external knee adduction moment. The magnitude of the first peak of the vertical ground reaction force was not significantly reduced but showed a noticeable trend when using the Pro-Flex foot (Fig. 5). The magnitude of force at the knee joint is highly correlated to the magnitude of the ground reaction force and this provides a proxy for how much force would be imparted with each step despite the debatable connection between magnitude of force and development of knee osteoarthritis^41,42^. The slope of the ground reaction force has been shown to correlate with sound limb knee pain^43^ and presence of knee osteoarthritis^44^, yet our data showed no differences between prosthetic feet (Fig. 5). CoM energy change during collision has not been a focus of osteoarthritis literature in the past and its connection to knee osteoarthritis is currently unknown. Yet, it does provide a measure of the total energy absorbed by the sound limb during early stance and should provide a reasonable measure of the impact on the sound limb. We did not demonstrate a significant difference between the prosthetic feet for CoM energy change during collision of the sound limb in an absolute sense, yet again, this also coincided with low statistical power (Table 1). Taken together, these data do show some promising trends but the low sample size precludes a definitive assessment on the effect of the additional energy return from the Pro-Flex foot to sound limb loading and the correlates to knee osteoarthritis development. These data should be used to predict adequate sample size for future studies.

In conclusion, the novel linkage system in the Pro-Flex foot does enable it to have more range of motion and better conform to different slopes. The Pro-Flex foot was able to leverage its additional range of motion to absorb and return more energy than the more traditional Vari-Flex foot. The additional energy return from the Pro-Flex foot translated up the kinematic chain to effect the whole body CoM in such a way that it enabled more energy for propulsion than the Vari-Flex foot. There was a significant inverse relationship between increasing CoM energy change during propulsion from the amputated limb and negative work absorbed by the sound limb during collision with the Pro-Flex foot and this gives some support that the Pro-Flex foot has promise to reduce sound limb loading. However, there were no other differences in other variables that correlate with reducing the risk of developing knee osteoarthritis, most likely due to the low sample size and low statistical power.

## Methods

### Subjects

Five people with a uni-lateral transtibial amputation (80.5 ± 13.9 kg, 1.73 ± 0.08 m, 44.0 ± 13.9 y/o, 11.2 ± 5.3 years post amputation) provided informed consent to participate in this study. The experimental protocol was approved by the Institutional Review Board at Alabama State University, (Montgomery, Alabama, USA) and these methods were carried out in accordance with the IRB-approved protocol. Inclusion criteria included having a uni-lateral transtibial amputation, the cause of amputation was not associated with a dysvascular disease, the individual regularly used a prosthesis for ambulation and could demonstrate variable cadence, be at least 2 years post-amputation, and use an energy-storage-and-return (ESR) prosthetic foot made from carbon fiber laminate on their everyday prosthesis. Exclusion criteria included having a cardiovascular or balance problem that would prohibit individuals from walking on a treadmill, and any muscle paralysis or loss of sensation in their amputated or sound limbs.

### Prosthetic feet

Two different types of passive ESR prosthetic feet were used in this study. The Vari-Flex foot (Össur EHF, Reykjavik, Iceland) represented a traditional design for an ESR prosthetic foot with a J-shaped carbon shank/forefoot section attached to a prosthetic heel (Fig. 1). In fact, the Vari-Flex foot is a direct descendant of the Flex-Foot design (Össur EHF, Reykjavik, Iceland) that was the first ESR brought to market in 1984^45^, defined the ESR class of prosthetic feet, and has been used in multiple studies on ESR foot performance^37,46–49^. The Pro-Flex (Össur EHF, Reykjavik, Iceland) represents a new design in ESR type prosthetic feet that includes a linkage system mounted between the heel section, the forefoot section, and the prosthetic pylon (Fig. 1). This design amplifies the deformation of the heel and forefoot sections to provide additional range of motion between the shank and foot segments during gait, and this should lead to greater energy absorption in the forefoot section which should translate to greater energy returned and ultimately more propulsion from the prosthetic limb during the pre-swing phase of gait. The stiffness of each prosthetic foot used were determined by the subject’s mass and activity level in accordance with the manufacturer’s specifications. Each prosthetic foot was ordered specifically for each subject and all utilized a 10 mm heel height. The subject’s prosthetic socket and suspension system was retained for this study. The prosthetic foot and pylon were replaced with the study ESR feet. Prosthetic alignment was initially setup according to specifications from the prosthetic foot manufacturer and then fine-tuned via clinical dynamic alignment for each foot. All prosthetic modifications and dynamic alignments were performed by a certified prosthetist (first author). The dynamic alignment period took between 5–10 minutes per subject per foot. The order of the prosthetic feet were randomized.

### Experimental Protocol

The subjects walked at 1.1 m/s on a dual belt instrumented treadmill mounted on a 2-DOF motion platform within immersive virtual reality projected unto a large 180 degree sweep screen (GRAIL system, Motek Forcelink, Amsterdam, NL). The virtual reality made the subjects feel as though they were walking through a forest instead of walking on a dual belt treadmill. Visual flow was set to match treadmill speed. 1.1 m/s represented the average self-selected speed of all subjects that have participated in previous studies at Alabama State University^50^ and is similar to the self-selected speed of other individuals with a uni-lateral transtibial amputation^7^. The 2-DOF motion platform allowed for the treadmill to be tilted to 7.5 degrees up and down. The subjects performed a 5 minute warm-up period on the treadmill to become acclimatized to walking on a treadmill, the virtual reality, and the prosthetic foot. The trials began with collecting data at the level condition for 1 minute after they completed the warm up period. The order for uphill or downhill was randomly selected, then the treadmill was tilted to 7.5 degrees, the subject was given at least 2 minutes to regain steady state gait and data was collected for one minute. This process was repeated for the other terrain condition. The prosthetic foot was swapped, and the experimental protocol was repeated.

### Analysis: Lower Limb Mechanics

A twelve camera motion analysis system (Vicon, Oxfod, UK) captured lower-limb kinematics (100 Hz). Kinetics (1000 Hz) were collected with two 6-DOF force platforms mounted under the treadmill (GRAIL system, Motek Forcelink, Amsterdam, NL) the Human Body Model markerset^51^ modified with three additional markers on each shank segment to create 4-marker clusters necessary for the ankle and foot deformation analysis^52^. A fourth order bidirectional low-pass Butterworth filter was applied to the kinematics (6 Hz) and kinetic (15 Hz) data. Data was collected, synchronized, and gap-filled using Vicon Nexus 2.5 (Vicon, Oxfod, UK). Data were then exported to Visual3D 6 (C-Motion, Germantown, MD) to build the limb segment models, filter the data, and calculate joint angles, moments, and powers on the continuous data. Data was then exported to Matlab 2017 (Mathworks, Natick, MA) to calculate individual limb work, identify and remove steps where the right leg crossed over to left belt and vice-versa, normalize each stride to 100 percent of the gait cycle, average 15 complete strides together per subject per condition, build group averages, and export the data for statistical analysis.

### Analysis: Foot Angle

ESR prosthetic feet are made to deform in order to store and return energy. This deformation is non-linear and this makes it difficult to define rotation of the foot segment about the shank segment as a single axis^53^. To overcome this limitation, foot angle was defined as the angle between the foot segment, defined by a four-marker cluster on the toe, head of the 1^st^ and 5^th^ metatarsals, and heel of the subject’s shoe, and the four-marker cluster on the shank.

### Analysis: Prosthetic ankle and foot power

The non-rigid nature of an ESR prosthetic foot in combination with the lack of a fixed joint axis may limit the use of traditional inverse dynamics calculations to adequately quantify the amount of power absorbed or returned by the prosthetic foot^53^. We employed a unified deformable segment (UD) model to overcome this potential limitation to both the prosthetic and sound limbs^52,54^. The UD model does not assume the foot is a rigid body and calculates power below the four-marker cluster mounted on the rigid prosthetic socket^52,54^. The energy stored and returned during propulsion was calculated as the time integral during the pre-swing phase of gait then normalized to body mass. Work-ratio was calculated as the ratio of positive to negative work, signifying the proportion of energy that was stored and returned by the prosthetic foot.

### Analysis: Whole body Center of Mass Rate of Energy Change

Rate of mechanical energy change of the whole body center of mass (CoM) was calculated to define how each limb contributed to propulsion of the CoM and handled collision with the ground during initial contact/loading response^19^. This was done by first dividing the summed right and left GRFs by the subject’s mass to attain the acceleration of the CoM. Then integrating the whole body accelerations with respect to time in each direction within the coordinate system of the treadmill. The average velocity across each whole dataset (from the first full stride to the last full stride during each condition) was assumed to be the treadmill belt speed of 1.1 m/s in the anterior/posterior direction, and zero for medial/lateral and superior/inferior directions. Individual limb rate of energy change was then calculated as the dot product of the individual limb GRFs and the CoM velocity, then normalized to body mass. The individual limb energy change during the step-to-step transition was the time integral during double limb support and single limb support were then calculated as the time integral of the rate of energy change during double limb support and then normalized to body mass.

### Statistical Tests

SPSS v23 (IBM, Armonk, North Castle, NY) was used to perform the statistical analyses. Pearson’s correlation was used to determine if a significant relationship existed between increasing ROM and prosthetic foot propulsion, energy returned during pre-swing and CoM energy change by the prosthetic limb, CoM energy change during propulsion by the prosthetic limb and CoM energy change during collision by the sound limb. A two factor RM ANOVA (foot x terrain) evaluated statistical differences (p ≤ 0.05) between feet within the group with an amputation. A mixed ANOVA (within subjects factor of foot x terrain and between subjects factor of limb) was used to compare the sound limb to the amputated limb. Mauchly’s Test of Sphericity was used to test if the variance was significantly different across all of the conditions. If the sphericity condition was violated, a Greenhouse-Geisser adjustment was applied. When a significance effect was detected with either the repeated measures or mixed ANOVAs, pairwise comparisons using a Bonferroni adjustment were performed to determine which conditions were significantly different. Effect size was estimated using partial eta squared (η_p_^2^) and any value greater than 0.14 would be considered a large effect size. Observed power was calculated as 1 − β.
